# The Out-Patient Registration Fee System

**Published:** 1886-11-06

**Authors:** J. C. Steele

**Affiliations:** Superintendent of Guy's Hospital


### The Out-Patient Registration Fee System.

By J. C. Steele, M.D.,
Superintendent of Guy's Hospital.

A Protective Tariff on Medicine.

Although agreeing with nearly everything that Lord Leigh has to say on the necessity of incorporating the provident principle with all systems of hospital organisation, I have a great aversion to the term Registration Fee being associated with it. It is no doubt a mistake to infer, because a protective tariff is employed for the advantage of the hospital as well as for the moral benefit of the patient, that it must detract from the charitable character of the former, but the mere mention of the word fee indicates a commercial relation with the higher objects of the charity which it would be wiser to avoid. Then, again, it is scarcely fair to the medical officers of hospitals, who for the most part give their services gratuitously, to prescribe for out-patients, to utilise their professional abilities in a way which they probably never contemplated, and which would possibly have the effect of bringing them into invidious competition and comparison with provident and other kinds of self-supporting dispensaries. It is far better to impose a nominal charge for value received in the shape of medicines and dressings, than to leave the applicant under the impression that he has the privilege of seeing the doctor for a sixpence or a shilling at any time. It is very likely that the doctor would recommend the patient to avoid indulging in medicine, in which case, as well as for all consultations, it is not at all expedient that any charge should be made. I should also be inclined to adopt a uniform sum in payment for physic, and to make it compulsory—that is to say, not to leave it to the option of the patient to contribute according to his own notion of his ability, but to refuse to continue supplying him with medicine on the score of poverty, unless he can give substantial proof that the few pence required of him by the laws of the hospital are beyond his means. The voluntary offerings of grateful patients have been pretty well tested in most hospitals, but the contributions found in the subscription-boxes in out-patient waiting-rooms have never been abundant enough to warrant any expectations of better results.

The Fee System at Guy's.

Compulsory methods of benefiting the charities, either by registration fees or by moderate charges for physic, have been in operation for years in some of the smaller London hospitals, but the plan was never enforced in any of the larger until two years and a half since, when, partly from a failure in its revenue, and partly also from a sense of its moral expediency, the Governors of Guy's Hospital introduced it among the out-patients attending that institution. The departure was better received at the time than I was prepared to expect, although the safeguards taken to prevent imposition on the one hand, and honest poverty from suffering on the other, relieved us of any anxiety with regard to results. But perhaps I had better explain the working of the system. Intimation was posted in the waiting-rooms, months beforehand, that on and after a certain date all out-patients would be required to pay threepence each time they received their week's supply of medicine, and if unable to pay, they should have their first supply gratis, but on the occasion of their second visit they should be provided with a certificate from the Secretary of the Charity Organisation Society of the district in which they resided, declaring them to be fit subjects for pecuniary relief. For the first few months after the innovation there was little perceptible difference in the numbers attending, but after a time they became considerably less, and the congestion and overcrowding which was formerly a marked feature of the department disappeared. The falling off was traced mainly to applicants suffering from surgical complaints, and greatly among such as had contracted venereal affections—a class which had hitherto formed the bulk of the surgical out-patients. On the other hand, the medical patients rather increased than otherwise, and rendered it necessary on at least two days of the week to send a small number away without being prescribed for. The drawings, which at first amounted to about £15 a week, have remained pretty steadily at nearly £14 weekly during the last eighteen months, and there is some reason for supposing that the number of patients is again on the increase. The money, however, can scarcely be considered to represent an equivalent gain in diminishing the cost for drugs, but it is an earnest of the goodwill, and possibly the gratitude, of the patients, who have always the option of going to other hospitals where there is no prohibitory tariff in force. Nor is there any reason for believing that the out-patients frequenting Guy's Hospital are of a better class than is to be found in other establishments of a kindred nature.

Social Condition of the Patients.

I have taken the trouble from time to time to make an analysis of the social condition of the applicants, and find that barely six per cent. express their inability to pay the nominal charge requested from them. Of this number about a third only have had their cases gone into by the local agents, and in about half of those investigated, the applicants have been deemed worthy of pecuniary assistance, while the remainder and the two-thirds who rejected the inquisitorial inquiry of the Society, have either remained away from the hospital altogether, after their week's supply of medicine was exhausted, or paid their pence without demur on the occasion of their subsequent visits.

Those Difficult Casualty Cases.

So far, it may be inferred that the provident, commercial, mutual accommodation, or by whatever name the self-help system may be known, has been on the whole satisfactory; but it has been found more difficult to deal with another always increasing class in all general hospitals, and to which, by common consent, the somewhat dubious term of casual is constantly applied. These consist for the most part of minor accidents, caused usually by their own or their neighbours' indiscretion, together with a variety of medical and surgical ailments requiring immediate relief from suffering, but not sufficiently serious to be received into the wards. They number from fifteen to twenty thousand annually at Guy's Hospital, and as the treatment mainly consists of some trifling surgical operation or the immediate application of splints, bandages, or other dressings, it is hardly possible, under the circumstances, to enforce a money payment in any shape, however advisable it may be thought to do so. In most general hospitals the department referred to is open day and night, while the out-patients' rooms, properly so-called, are limited to a few hours of the forenoon or afternoon; and it is quite possible that the facilities afforded by the former for escaping payment may have led to the very perceptible increase of patients in the department.

Self-help and Free Relief.

It may be sentiment, if not the matured opinion of many, that medical charity, like the Gospel, should be freely dispensed without money or price; but, in the face of the greatly improved provision made for the legalised poor, and the difficulties experienced in obtaining the necessary means for the maintenance of voluntary charities, it is high time that the recipients should be made to feel the necessity of self-help in a matter which so intimately concerns them. The supporters of hospitals are bearing an ever-increasing burden, and if it be true, as has been repeatedly stated, and never contradicted, that the subscription lists of the various London hospitals (apart from vicarious Saturday and Sunday collections) represent only one in a thousand of the population, it is but fair that the select few should be relieved, to however small an extent, by the people whom they are befriending.

